# Attitudes of Young Tri-City Residents toward Game Meat. Development and Validation of a Scale for Identifying Attitudes toward Wild Meat

**DOI:** 10.3390/ijerph20021247

**Published:** 2023-01-10

**Authors:** Dominika Mesinger, Aneta Ocieczek, Tomasz Owczarek

**Affiliations:** Faculty of Management and Quality Science, Gdynia Maritime University, 81-225 Gdynia, Poland

**Keywords:** game meat, attitudes, scale validation, reliability, pre-test sample, pilot study

## Abstract

Attitudes toward food are one of the most critical factors related to consumer behavior in the food market. Therefore, identifying attitudes toward a specific food product may be essential for identifying factors influencing certain behaviors regarding game. In addition, game meat is a valuable food that can increase the variety of meat and reduce the intensive breeding of slaughter animals. Therefore, a research gap was found regarding the lack of a tool for identifying attitudes toward game that would allow for the acquisition of data valid for studying conditions related to game consumption. The study aims to validate a developed scale for identifying the attitudes of young Tri-City residents toward game. To collect the database, two groups of respondents are involved in the validation procedure. This procedure includes validation of content, response process, and statistical validation. The scale is validated, and four domains are distinguished based on the PCA test. The validated scale consists of 10 statements (initially 11). The estimated Cronbach’s alpha (0.6944) indicates good scale internal consistency. The developed scale can be used to identify attitudes of young Tri-City residents toward game and search for links between these attitudes and behaviors related to game consumption.

## 1. Introduction

### 1.1. Game Consumption in Poland and the World

For thousands of years, the meat of wild animals has been the fundamental source of wholesome protein and other nutrients and a way to diversify the plant food base of man. As humankind developed about 10,000 years ago, the acquisition of meat took the form of the taming and domestication of wild animals. Such action allowed man to ensure continuous access to food of animal origin. Selected breeds of animals were selected and bred. As a result, the production of raw materials of animal origin (meat, eggs, milk, and skins) of the best quality was intensified. Despite these efforts, hunting still functions in all countries of Europe and the world, although in different forms, for different purposes, and for fulfilling different functions [1].

In this article, “game meat”, “game consumption”, “attitudes toward game”, as “game” should be understood according to the literature of the subject all wild animals that were obtained by hunting. These are, therefore, among others, wild boar, red deer, roe deer, fallow deer, pheasants, partridges, hares, etc.

Game meat should undoubtedly be considered a traditional product because, in the culture of Poland and Europe, it appeared in particular at the courts of kings and nobility. Nevertheless, the tradition of consuming this meat gradually disappeared, to such an extent that the younger generations now consider it as a new, innovative product. This state of affairs is referred to in the literature as the secondary product life cycle. Therefore, it should not consider game-meat-related marketing activities through the prism of the standard life cycle of a product, in which there are four key stages: market launch, increase in interest, market maturity and saturation, and decline leading to the death of the product [2]. In the case of game meat, this pattern, instead of the death of the product and its withdrawal from the market, includes the re-development of the product and the continuation of the remaining phases. Thus, since currently, game meat is a product in the first or second phase of the life cycle of a product, it can be treated by consumers as a new product.

In 2014, Schulp et al. [3] conducted a study on the amount of game consumed by humans in Europe. However, according to the nomenclature, the meat of all wild animals, among others, deer, fallow deer, roe deer, wild boar, hare, and wild birds, was considered as game. The study was conducted in 2014, but their results still seem valid in 2022. The highest game consumption was recorded in France, where it was 5.7 kg/person/year. High game consumption was also recorded in Italy, where it was 3.8 kg/person/year. However, there are countries such as Poland or Portugal where game consumption per capita per year was set at 0.08 kg. The lowest level of game consumption was recorded in Bulgaria, where it was 0.02 kg/person/year.

The total meat consumption in Poland per capita per year is approximately 72.9 kg. Of this, pork and poultry are the most significant contributors and, in small amounts, beef [4]. The government should consider what caused this situation because game is an extremely valuable product, and its acquisition is essential. Due to the constant increase in the number of wild animals and the simultaneous expansion of the limited resources of green areas as part of anthropological activity, it is necessary to acquire a certain number of wild animals constantly. Such action is justified because the amount of game must be adjusted to the size of the area it occupies. At the same time, the disposal of meat obtained in this way appears to be a waste, which is by definition something irrational and even ecologically harmful. Every year, the number of wild animals obtained in Poland remains relatively constant, amounting to about 100,000 deer and 200,000 roe deer, 300,000 wild boars, and 80,000 pheasants, which gives hundreds of thousands of tons of full-value raw material. Importantly, although Poles do not eat game, Poland is a huge producer and exporter of it. According to the Central Statistical Office estimates, about 50,000 tons of game are exported from Poland every year. The largest recipients of this valuable raw material are, among others, Vietnam (10,000 tons), Germany (7200 tons), Italy (4200 tons), and the Netherlands (3500 tons) [5].

Sourcing wild animals is undoubtedly a must, which means that game is a by-product of forest management. Hence, it would be an utterly non-ecological action to abandon or limit its management, which we are currently dealing with. This statement is essential, especially because the “farming” of wild animals complies with the guidelines defined for organic food because it is not dependent on humans in any way. Man does not have to feed wild animals; he does it only in the case of tough winters or when wild boars devastate the surrounding crops. Wild animals are not treated with antibiotics, hormones, or other chemicals. No particular places, machines, devices, or halls are needed to breed these animals. They live in the wild and can move freely depending on their needs. Therefore, also, such an element of their existence as the production of gases, which affects the pollution of the environment, is negligible. Of course, ruminants are also identified among wild animals, producing methane. However, due to their movement and small numbers, it does not accumulate in one place, as is the case with large-scale industrial livestock farming. For the definitional order it should be noted, however, that in a strict sense the meat of wild animals is not the same as organic meat [6].

Game is a nutritionally valuable product and, at the same time, has a sustainable environmental impact, so it should not be exported. The justification for this position is the consequences for the natural environment resulting from meat transport as an integral part of export. Meat intended for export must be frozen before the transport process, which causes environmental pollution. The transport itself, generating exhaust fumes, also increases the carbon footprint of this meat. Such burdens, both financial and environmental, are unjustified. Therefore, it is advisable to use this raw material in the country of harvest. Other losses related to the export of game meat include the reduction in its availability in Poland. This may result in higher prices for this meat and, at the same time, limit the base of nutritious bioactive ingredients (i.e., CLA, coenzyme Q10, taurine, carnosine, anserine), wholesome protein, and high-quality fat [7,8].

### 1.2. Attitudes toward Food and Their Neophobic and Neophilic Determinants

Game consumption and its conditions may result from the attitudes of consumers. In the literature, food neophobia is defined as an attitude toward food that manifests itself in the avoidance of eating new products unknown to the individual or a general reluctance even to try them. Therefore, neophobia is an extremely negative trait, and its opposite is neophilia. Neophilia is expressed as a general willingness to try new or previously unknown kinds of food [9].

So far, the highest level of food neophobia has been observed among children, and its level decreases with reaching adulthood. However, it happens fairly often that older adults show an increased level of neophobic attitude, which is conditioned by changes in the body and mentality of an aging person. In the literature, one can also find information on the determinants of food neophobia among adults. Although food neophobia is a genetically determined trait, it is estimated that heredity allows the explanation of the variability and the occurrence of neophobia in cases. On the other hand, the remaining one-quarter of cases result from environmental factors, particularly from the mother’s prenatal nutrition or diet and eating habits obtained in childhood. Other important factors that influence the level of neophobia include personality and lifestyle [10,11,12].

An excellent example of cultural differences in neophobic and neophilic attitudes toward food is the consumption of insects—entomophagy. According to the literature on the subject, it can be stated that the level of insect consumption, and even the approach to this issue, varies depending on the region of the world. In some Asian, African, and South American countries, insects are a traditional food item that is common in the normal diet. In most European countries, as well as in Australia, northern Asia, and the United States, insects are considered a kind of food taboo or “barbaric tradition” [13].

### 1.3. Attitudes toward Food and Their Relation to Eating Behavior

Therefore, the attitude is a hypothetical construct and cannot be assessed by observation [14]. However, it has also been shown based on a meta-analysis of 61 studies that overt attitudes strongly influence consumer behavior. Therefore, the identification of overt attitudes toward food, related to eating behaviors classified as consumer behavior, is based on the results of this meta-analysis [15,16].

According to the definition presented by Ajzen [17], an attitude is an individual disposition of an individual to react positively or negatively to an object, person, institution, event, and any other aspect of the world. However, it should be noted that this is only one of many definitions of attitudes that could be found in the literature. However, most of them assume that a characteristic feature of an attitude is its evaluating dimension, namely positive or negative attitude, for or against [18,19,20].

Thus, in line with this assumption, almost all techniques and methods of examining consumer attitudes allow for placing the result of a given individual on a continuum of evaluation in front of the attitude object [20]. It is also possible to analyze the attitude not as a general evaluative disposition but by analyzing the structure of the domain to which the attitude relates.

It should be noted that attitudes are not related to beliefs. They are a specific function of an individual’s beliefs about a specific object. Therefore, the concept of attitude is highly complex, and this is how it should be understood and considered. In general, beliefs are assumed to have a causal effect on attitudes. Every belief about a given object connects it with a specific attribute. On the other hand, the subjective value of an attribute influences the attitude in direct proportion to the strength of this belief [17].

One of the most important psychological factors that determine specific consumer behavior, including eating behavior, is the attitude toward the object of consumption. According to the literature, attitudes should be treated as the result of three components: cognitive, emotional, and behavioral [21,22,23,24]. The components of the cognitive component include knowledge about the attitude object, the way this object is perceived, and its memory connotation. The emotional component, also referred to as the affective component, indicates an attractive or repulsive feeling that individual experiences toward the object of the attitude. The last component of the attitude, namely the behavioral component, consists of intentions to behaviors, including the emotional relationship and knowledge of the subject of the attitude.

Considering the relations of consumers’ attitudes toward their behaviors, it should be emphasized that the attitude that has been identified in relation to a specific food does not have to be consistent with the consumer’s behavior toward that food. Indeed, factors described as mediating variables or confounders are likely to be effective in weakening the relationship between attitude and behavior. The disturbing factors include economic limitations, situational factors, such as the influence of accompanying persons while shopping, and subjective norms and environmental factors, including the influence of family members and the environment [25,26].

The above premises were the reason for undertaking research on identifying respondents’ attitudes toward game as a factor constituting the intention to preserve the potential for the development of the game meat market in Poland.

### 1.4. Review of the Literature on Attitudes and Behavior toward Game

When analyzing the publications on attitudes and behaviors toward game, it can be stated that many factors influence the final shaping of the attitude toward this meat. For example, in a study conducted in Poland by Czarniecka-Skubina et al. [27], consumers’ consumption of game is determined primarily by its taste. Some less important factors also included the health benefits of eating this meat, family traditions, and the presence of allergies in family members. As some of the respondents were hunters, they also indicated that large amounts of game are available to them, and they want to manage it somehow. On the other hand, the most common reasons for avoiding game consumption were the lack of tradition in eating it, low availability (among people not engaged in hunting), high price, fear of contamination with diseases, unacceptable taste, and inability to prepare it. Moreover, almost 25% of people who did not eat wild animal meat indicated ethical reasons and the will to care for the planet.

In a 2016 study, Tomasevic et al. examined the attitudes of 2935 meat consumers, asking them to state to what extent they agreed with various statements, e.g., game and an indication of why they eat it. The respondents included the most common reasons for the consumption of game: eating enjoyment, nutritional value, desired taste, but also juiciness, better nutritional values than other types of meat, and tradition [28].

In turn, in the research of Wassenaar et al. from 2019, it was found that the key factors influencing the attitudes toward game include such attributes as availability, sensory characteristics, ethics of obtaining meat from wild animals, but also the health benefits of eating this meat. It should be noted that these are general trends for the entire study population. However, there were differences in the choice of attitude determinants when respondents were divided into the group of meat consumers and the group of those who do not consume it. Meat eaters indicated that game had positive sensory features, and non-consuming ones indicated that game sensory features repels them. Thus, each of the factors mentioned above can be considered in two ways regarding the attribute of attitude [29].

The literature indicates many factors that consumers perceive as important for the safety of game meat. The most common threats are diseases such as trichinosis and toxoplasmosis, but so are genetic modifications of meat or the huge amount of hormones contained in it. A critical analysis of the literature data allows us to conclude that the above-mentioned factors do not increase the risk of game consumption. The literature on the subject even states that game meat is in many respects much safer than meat from farmed animals. Therefore, apart from specific sensory characteristics, certain myths prevailing in society undoubtedly affect the reluctance to consume game meat [30,31].

### 1.5. Purpose of the Research

A critical analysis of the literature showed substantive and research gaps in the identification of attitudes toward game. These attitudes have not been identified among young people who are the most susceptible to the absorption of novel food products that appear on the market, including those going through their secondary life cycle. Therefore, this study aimed to develop and validate a scale enabling the identification of attitudes of young people from the Tri-City toward game meat.

## 2. Materials and Methods

### 2.1. Selection of the Sample of Respondents for the Main Study

The main study was conducted in 2019 among 453 young inhabitants of the Tri-City aged 18–40. The selection of young people aged 18–40 as a research sample was conditioned by the fact that, according to scientific reports, it is a group of consumers characterized by low consumption of game meat. At the same time, it is a group susceptible to changes in eating behavior, looking for new products, and sensitive to changes in the environment or climate, and one that is insufficiently researched in terms of attitudes toward game meat and their relationship with the consumption of this raw material. According to the premises contained in the literature, it was found that generation Y (born in the 1980s and 1990s) and generation Z (born after 1995) will constitute the research sample because of their highest activity on the food market. It was found that people aged 18–40 are the most active when it comes to making purchasing decisions, but also this age discrepancy allowed us to capture differences in consumer attitudes to a wider extent. People from the age of 18 were selected because it was assumed that they are consciously and independently making food choices because they are in the age of majority. In addition, people over the age of 18 were often students, who make up a significant proportion of the population in the study region. On the other hand, the limit value, which was the age of 40, was considered final because, according to the research conducted by the Public Opinion Research Center in Poland, the average age begins after the age of 40 [32,33,34]. The authors wanted to conduct research using the PAPI method, which is why the nearest agglomeration, which is the Tri-City, was selected. This is an area that, to some extent, may show various tendencies prevailing in the country because of the significant number of immigrant inhabitants. Large numbers of them are conditioned by the seaside location, but also by the presence of numerous, very popular universities in the country. The Tri-City agglomeration consists of three cities that are extremely different from each other. Namely, Gdańsk is a large and very developed city, Gdynia is a medium-sized and developing city, while Sopot is the smallest city and, above all, a tourist city. People participating in the study were randomly selected from among people living in the Tri-City (northern Poland). A stratified random selection of the test sample was used. The study area was divided into three layers concerning cities: the first—Gdańsk, the second—Gdynia, and the third—Sopot. Two Biedronka stores, one located in the city center and the other on its outskirts, were selected to conduct the study in each tier. As a result, the research involved young people living in the Tri-City who take an active part in shopping for groceries and therefore demonstrate specific consumer behavior and exhibit specific attitudes toward selected or rejected goods. Therefore, the main study sample was not a representative respondent sample of the population of young people living in Poland.

The survey was conducted in an anonymous form, following the guidelines of the Helsinki Declaration. Therefore, apart from anonymity, the study also considered ethical standards and the respondents’ rights, and so on [35].

### 2.2. Selection of a Sample of Respondents for the Initial and Pilot Study

The data to validate the scale were collected in 2019, prior to the main study. The validation procedure involved collecting data in two different samples of young people from the Tri-City area (Poland) in the pre-test and pilot samples.

The study was carried out in northern Poland, in the agglomeration known as the Tri-City, including Gdynia, Sopot, and Gdańsk. It is an agglomeration that represents large cities in Poland very well and, at the same time, is characterized by a significant diversity of the population related to, among other things, the fact that in these cities there are numerous academic centers. Each year, many young people from all over the country come to the Tri-City to study at the largest universities in the region. Moreover, each of the cities included in the agglomeration is definitely different in terms of the number of inhabitants, so it can be assumed that this will also reflect the general attitudes that characterize people from various types of larger cities. The Tri-City is an intensively developing agglomeration with many innovative initiatives.

The pre-test sample consisted of 10 respondents, 6 of whom were women (60%). Data on variables other than gender have not been assessed because of the low influence on the validation results. According to Yusoff [36], the minimum number of participants in the validation of the response process should be 10, although it was usually 30 respondents in most validation studies. Therefore, in this study, the minimum sample size criterion at this stage of the validation procedure was met at a minimum level.

The pre-test participants were involved in validating the response process, while the pilot sample participants were involved in the statistical validation study. People from both trials were residents of the Tri-City, but they were not the same people. This procedure was used to avoid the impact of knowing the scale statements on the answers to the main study.

The number of participants in the pilot study was determined according to Hair et al.’s statement that the minimum number of respondents in this type of survey is five for each statement that builds the scale [37]. Due to the fact that, after validating the content and validating the response process, the scale of identifying attitudes toward game consisted of 10 statements, the survey should cover at least 50 respondents. The pilot sample consisted of 453 people. Thus, the condition of the minimum sample size was met. When evaluating the gender data, it was found that women accounted for 69.75% of the pilot sample.

### 2.3. Procedure for the Development and Validation of the Scale for Identifying Attitudes toward Game Meat

The 5-point Likert scale was used in the questionnaire. The use of a 5-point scale is conditioned by the perception of the human mind. Miller [38] found that the human mind has limits on the amount of data it can process. That study found that the number of different answers to choose from within 7 ± 2 is the most justified. Hence the superiority of the 5-point Likert scale over other scales (3-, 7-, or 9-point).

In the Likert scale, category labeling is used, which means that apart from digital markings, e.g., 1–5, individual categories are assigned names that help the respondent indicate the degree with which he or she identifies the most. In this questionnaire, the most basic differentiation of the Likert scale categories was used: −2—I disagree completely, −1—I disagree, 0—I do not care, 1—I agree, 2—I completely agree. However, using a scale that is too long in the present case could lead to a tendency to flatten it, i.e., combine several points on the scale into one [39].

When creating a scale for identifying attitudes toward game meat, the knowledge obtained from the literature review on the acquisition and consumption of game was used (Table 1). The research instrument consisted of 11 statements, including 6 positive and 5 negative. Positive and negative statements have been randomly placed to prevent the influence of the respondents’ thoughts. The scale developed in this way was validated, and validity was understood as the degree to which the scale measures what it is supposed to measure [40]. This was followed by a validation procedure with four main steps:

1. Content validation assesses how individual elements of the assessment tool (statements) are relevant and representative of the tool serving the defined purpose of the assessment. At this validation stage, it is necessary to involve expert judges, whose task is to evaluate the content of the statements that build the scale [41].
2. Validation of the response process (the so-called face validation) is used to assess the relationship between the intended construct consisting of a set of statements and the mental processes of people participating in the study. This validation stage is usually carried out on a small sample of respondents representing the final test sample [42]. Statistical validation was carried out based on the results obtained in the pilot study. In this study, respondents referred to a set of statements using a 5-point Likert scale ranging from “strongly disagree” to “strongly agree”, which were assigned a number of points reflecting the increasing intensity of approval of the statement. The scale coding from −2 to 2 with a neutral value of 0 was used. This procedure allows for the recognition of such a constructed assignment as a feature on an interval scale. It enables the performance of basic mathematical operations, particularly the mean and standard deviation calculation. However, it should be remembered that this is a kind of approximation or simplification, and it is necessary to carefully interpret the obtained values [43,44]. The negative statements were recoded, i.e., the ratings for individual answers were reversed (statements 2, 5, 8, 9, 11—Table 1), giving −2 points for the answer “I strongly agree” and 2 points for the answer “I strongly disagree”. Statistical analysis was performed using the Statistica 13.0 PL software (StatSoft). The identified differences were classified as statistically significant at *p* ≤ 0.05. Statistical validation, also called construction validity, was performed using exploratory factor analysis (EFA) to achieve the comprehensibility and unambiguity of the new scales [37]. This procedure takes two steps: 3a. preparation of descriptive statistics taking into account each statement’s mean values and standard deviations, and 3b. conducting principal factor analysis (PCA) using varimax orthogonal rotation. Before performing principal components analysis, the KMO (Kaiser–Meyer–Olkin) statistic was calculated, and the Bartlett sphericity test was performed. A KMO value greater than 0.60 indicates that the data are appropriate for factor analysis. On the other hand, the Bartlett sphericity test allows the determination of the correct distribution of the test sample. Therefore, factor analysis can be performed if the distribution of the test sample is correct [45].
3. Scale reliability testing used Cronbach’s alpha based on data from a pilot study. Scale reliability is how the tool used can be expected to give the exact measurement results when the study is repeated [40].

When justifying the choice of the method of conducting statistical analysis, it is worth emphasizing that PCA is an analysis widely used in the validation of questionnaires related to food [46]. In turn, the KMO test and the Bartlett sphericity test were used in the validation of the scale concerning the emotional intelligence of consumers [47]. However, it should be noted that this is only one of many concepts for validating the consumer assessment construct. There are many others as good as, for example, primary and secondary validation used by Granbois and Summers [48] in a study on the probability of purchasing a given product.

## 3. Results

The first step of the validation procedure involved checking the content, i.e., validation based on interviews with a panel of four experts in the field of assessing attitudes toward food products. After the content was validated, the statement, “Nowadays, hunting is a pastime, not a necessity,” was removed from the scale, which in fact refers to hunting, not game. However, there are reports indicating the perception of meat by consumers through the prism of how it is obtained. In this case, it was concluded that this statement would disturb the thought process of the respondents. It should be emphasized that attitudes toward meat, not only game, are formed by cognitive, emotional, and behavioral factors. Attitudes are related not only to the quality characteristics of meat but also to its production method and the impact of production on the environment.

The second stage of the scale validation was the validation of the response process, which was aimed at assessing whether the individual words, as well as the sentences used to create the statements appearing on the scale, are easily understood by the respondents. Therefore, respondents were asked to underline words or sequences in individual statements, the meanings of which were not fully understood by them. The scale did not contain any incomprehensible statements according to the collected data. Consequently, this validation stage did not cause any changes in the created scale. The validated research instrument contained 10 statements, including 6 positive and 4 negative.

The lowest average (−0.41 ± 1.1301) was recorded for statement 6—“The nutritional value of wild animal meat is the same as that of farm animals.” This means that respondents do not see any clear benefits associated with its consumption. However, the highest mean (1.02 ± 1.0702) was recorded for statement 5—“Eating wild boar meat is synonymous with trichinella infection” (Table 1). This result was obtained after the responses were recoded, which means that the respondents were aware that this statement was untrue.

After the content and response process were validated, statistical validation was performed using exploratory factor analysis. This analysis first included the Kaiser–Meyer–Olkin (KMO) test to determine the adequacy of the sample selection for factor analysis [47]. The KMO measure takes values from 0 to 1, and the greater its value, the more reasonable it is to reduce dimensions by means of factor analysis. The value of 0.5 is usually taken as the limit of such validity. The KMO value for the examined set of statements was 0.71, which should be considered definitely sufficient to reduce dimensions and appropriate for factor analysis.

Second, the Bartlett sphericity test was performed to check whether the data from the pilot trial were appropriate. The results of this test also confirmed the applicability of factor analysis. The test checks whether the correlation matrix between the variables is a unit matrix (null hypothesis of the test) [47]. In such a case, the use of factor analysis would be unjustified because of the lack of a significant relationship between the variables. For the analyzed set of statements, the value of the test statistic χ^2^ in this test was 1515.2, which allowed the rejection of the null hypothesis at any significance level. On this basis, the validity of factor analysis was confirmed.

**Figure 1:**
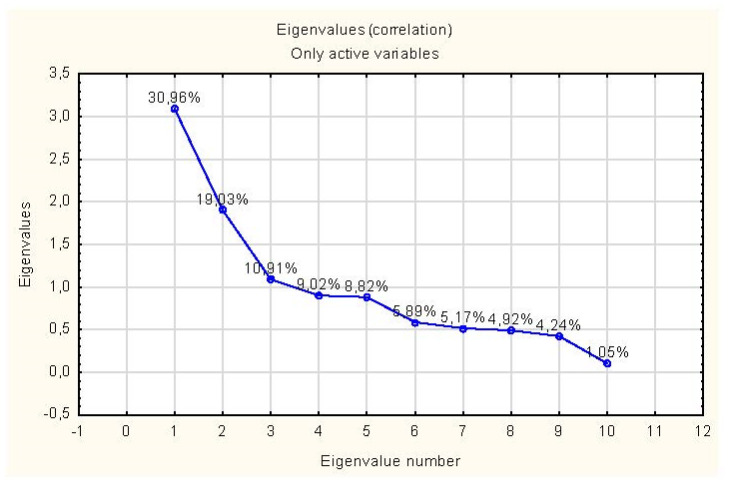
Scree plot.

The third step of the statistical validation procedure defined the necessary number of reduced dimensions (domains). For this purpose, one can use the Cattell criterion using the scree plot, the Kaiser criterion (eigenvalues greater than 1), or a criterion based on the cumulative variance of the analyzed variables (see Table 2 and Figure 1) [49]. Assuming that the Kaiser criterion indicates too few factors for a small number of variables (less than 20), it was assumed that four factors would be appropriate for further analysis.

To better interpret the obtained factors, it was decided to introduce the varimax rotation. As a result, the factor loadings (correlations between the factor and the observed variable) for each variable have large values for only one factor and are relatively small for other factors. This makes it easier to assign variables to the factors describing them [50]. The values of the factor loadings are presented in Table 3. The load value equal to 0.6 was adopted as the limit of linking the factor with the variable.

As a result of this procedure, four domains were built. The first was related to statements 1, 3, and 4. It described game meat as a food suitable for everyone but not necessarily available. Domain 2 was associated with statements 2, 5, 6, and 8 related to the possible risks or no benefits of game consumption. Domain 3, related to statements 7 and 9, described the characteristics of game meat that distinguished it from other meats. In turn, domain 4 indicated difficulties in preparing this meat for consumption.

The resulting domains were also tested for reliability. The estimated values of Cronbach’s alpha are presented in Table 4.

Cronbach’s alpha for domains 1 to 3 took a value greater than 0.6, which should be considered satisfactory. However, for domain 4, Cronbach’s alpha was not calculated because there was only one statement in this domain.

Then, the values of basic measures of descriptive statistics were calculated for all variables and domains. For this purpose, the values of means and standard deviations were estimated as measures and also used in factor analysis. Medians and quartile deviations were also calculated as measures more appropriate for the description of observations on the Likert scale. However, the medians are not very interesting measures as they assume almost the same values in all cases, i.e., 0. The results are summarized in Table 5.

Much more interesting results were obtained by analyzing the behavior of the quartiles. In the first domain, the first quartile was −1, and the third was equal to 1. This means that at least half of the respondents referred to the statements of this domain, indicating answers between −1 and 1. This domain shows a practically perfect neutrality of responses. There is practically the same number of positive and negative answers. The average value of 0.03 confirms these observations. The respondents have an ambivalent attitude to the issues raised in this domain.

For domain 2, the first quartile is −2, and the third is 0, the same as the median. This means the answers of the evaluators to 0 were the most numerous; at least 25% of the answers indicated −2. A large group of respondents strongly disagreed with the statements of this domain. This is confirmed by the domain average of −0.6. The respondents primarily do not see the safety risk that comes from game meat consumption. Although, for the most part, they do not have an opinion on this matter, it can be noticed that there is a need to constantly educate people about the safety of this meat. Perhaps reassuring them that game is safe will encourage more people to try it.

The median in domain 3 is equal to 0, and the mean value is equal to 0.07; this indicates that also, in this domain, neutral responses outweigh the others. In this case, however, the value of the first quartile is 0, and the third is 1. Half of all responses are within this range, and 25% are responses of 1 or more. This indicates a slight shift toward responses in line with the domain statements. The respondents have relatively neutral attitudes, but sometimes they tend to believe in the positive nutritional value of game meat. Thus, the respondents were at least partially aware of the positive qualities of game: its low amount of fat and high content of minerals.

In domain 4, as in domain 3, the average values indicate a neutral attitude of the respondents to difficulties in preparing game meat. However, the mean value equal to 0.24 (the highest value among the domains) and the quartile values (the first one is 0 and the third one is equal to 1) indicate that difficulties with preparing game meat may be significant for many people. So, this could make contact with this type of meat difficult and, at the same time, discourage them from trying it at home.

## 4. Discussion

Attitudes are believed to be among the main factors influencing behavior, including eating behavior such as eating new or little-known foods. Considering the fact that game, despite its many undeniable values, is not very popular among young people in Poland and the fact that increasing its consumption in the place of its acquisition would contribute to both increasing the variety of food available on the market and diversifying the diet, as well as bringing tangible benefits for the environment and climate, it seems justified to define attitudes toward game meat in the group of young people. Identifying attitudes toward wild animal meat would make it possible to search for links between these attitudes and variables such as gender and socioeconomic factors. Furthermore, it will help in searching links between attitudes and behaviors related to game consumption. However, there are substantive and research gaps in this respect. Therefore, a scale research tool was developed to identify young people’s attitudes toward game. The constructed scale has been validated.

Before developing the scale, the literature on game meat consumption in the population of young people in Poland was analyzed. Based on the information obtained from this analysis, a scale consisting of 11 statements was constructed. The scale contained a comparable number of positive and negative statements. The authors first resolved the doubt as to whether it was more advantageous to develop a scale with more or fewer statements. The conclusion from this decision was that using a smaller number of statements would allow us to obtain a tool that allows us to receive essential data about the attitude object and, at the same time, to use it efficiently. It is well-known that potential respondents are reluctant to participate in a survey if it is associated with the need to devote a long time to it.

When compiling the lists, the knowledge about the positive and negative aspects of game consumption and society’s prevailing beliefs was used. Among the aspects of game consumption that can be considered positive, there are such inherent properties of game meat as health benefits resulting from its inclusion in the diet, a nutritional value comparable to slaughter animals. Moreover, at the same time, game meat consumption is more beneficial than livestock meat. Game meat is lean and has a high amount of minerals [8]. These statements were mainly related to the cognitive component of attitudes, i.e., knowledge, opinions, and beliefs about the properties of game. Other positive statements indicated that game meat would be readily bought if it were cheaper and widely available. These statements can be considered as elements related to the behavioral component of the attitude to game meat.

Claims related to the negative aspects of game consumption included references to the health risks of game consumption (high levels of heavy metal contamination of this meat). This statement is functioning in the information space, although there are many scientific publications indicating that it is only a frequently repeated myth [6]. Therefore, this statement was used to express negative opinions and emotions toward game. Among other negative statements that fit into the cognitive component, one was proposed related to the difficulties in the culinary preparation of game meat. Moreover, among the negative statements of an emotional nature, one that has been proposed stated that game meat is poorly researched and therefore little-known. In this case, it can be concluded that the emotional component is connected with the cognitive component of the attitude. The perceived lack of knowledge about the health risks associated with eating wild animal meat may result in the expression of negative emotions toward this meat [51]. Among the emotional components of the attitude was a statement that did not refer directly to game but to obtaining it. Numerous studies showed that consumers reject the possibility of eating game only because it is obtained from hunting. This method of killing animals is perceived as inhumane. However, it is not true because it does not involve greater suffering for animals than in the case of industrial slaughter and even less ritual. The last statement expressing a negative attitude toward game was behavioral and was associated with the risk of trichinosis infection when consuming wild boar meat. It is true that eating raw wild boar, like raw pork, is unacceptable because of this risk.

To assess the accuracy and credibility of the constructed scale and consequently to achieve the goal set in this study, which was the identification of attitudes toward game meat, it was validated. Validation is an essential procedure in developing research tools to measure specific constructs such as attitudes [37,52]. Therefore, the developed scale was validated through content validation, response process validation, statistical validation, and plausibility testing based on Cronbach’s alpha calculation. After the content of the statements building the scale was validated based on experts’ opinions on attitudes toward game meat, the number of statements was reduced from 11 to 10 items. The statement removed from the scale referred directly to hunting, not game. The literature review shows that other researchers also involved a panel of experts at the start of the validation procedure for the new scales [53]. Expert opinions were used to modify or reject scale-building statements.

The following validation step consisted of validating the response process, which showed that respondents had no difficulty understanding the meaning of the game meat attitudes scale (GMAS) statements. This step did not necessitate any changes to the scale-building statements. However, this step was necessary to ensure that the statements were understood by any ordinary respondent. In the situation in which even only one of the statements is misunderstood, the respondents will give answers that are inconsistent with their factual beliefs, which, when analyzed comprehensively, will lead to false conclusions. Therefore, it should be emphasized that the validation of the response process does not eliminate but only minimizes the risk of misunderstanding the statements by the respondents. Therefore, this step should not be omitted during the validation procedure of a new research instrument.

The results of the principal component analysis (PCA) performed with the varimax rotation allowed identifying four domains. Therefore, it was assumed that the scale identifying attitudes toward game should be divided into four independent subscales that describe attitudes toward specific aspects related to game. The analysis of the nature of the statements showed that the first subscale would consist mainly of positive statements. In contrast, the second subscale would consist of negative statements and did not distinguish game meat from farm animals’ meat. Therefore, the use of such subscales to identify attitudes toward game would be unjustified because the scale for identifying attitudes should include positive and negative statements, and the number of such statements should be similar. When analyzing the structure of other scales developed and verified to identify attitudes toward food products, it can be concluded that some of them consisted of several subscales.

An example is a scale for assessing attitudes toward chocolate, consisting of three subscales: guilt, emotional eating, and chocolate cravings [54]. Another scale for identifying attitudes related to health and taste has been divided into six subscales measuring individual domains of attitudes. Contrary to the subscales distinguished in this study, each of these six subscales contained the same number of negative and positive statements. However, this is not a defect of the validated scale but its specific characteristics [55].

The last step in the validation procedure was to calculate Cronbach’s alpha and it did not remove any of the statements from the scale. The research instrument developed and validated in this way can be considered balanced in terms of the nature of the statements. The Cronbach’s alpha value (0.6944) indicated high reliability of the scale in identifying attitudes toward game. Thus, the proposed scale identifying attitudes toward game meat was accurate and was characterized by good internal consistency.

## 5. Conclusions

The developed and validated scale can be used to identify attitudes toward game in the population of young people. Identification of attitudes toward game meat may be of significant importance for managing the hunting economy and the promotion of health. Furthermore, it would be part of the trend of research supporting the idea of sustainable consumption, which is part of the green deal. The use of such an instrument can therefore contribute to progress not only in the area of social sciences related to management and quality sciences but also in the area of health sciences and life sciences.

## 6. Limitations of the Research

The subject of respondents’ attitudes toward game meat is undoubtedly very interesting, but also complex, which is why the study, like many others, has its limitations. Undoubtedly, the biggest limitation of the study is the limited population and area of the study. According to scientific reports, it can be said that attitudes are something variable, so even if the study was carried out again on the same people in the same places using the same questionnaire, but at a different time, the results would certainly be different. That is why it is so important to validate surveys, because it allows you to check their reliability.

Another limitation of this study is the age of the respondents, gender, and other socio-demographic characteristics. As a result, the research is not representative of the entire study area. In addition, only some sociodemographic data were analyzed in the metrics of this study, which may also be a limitation because a larger amount of socio-demographic data obtained from the respondents would allow for a deeper analysis.

## Figures and Tables

**Table 1:** Description statistics of the list of statements for identifying the attitudes of respondents toward game obtained in the pilot study.

No.	Statement	Average Value	SD
1.	There are health benefits to consuming wild animal meat.	−0.02	±1.1539
2.	Wild animal meat is heavily contaminated with heavy metals. (N)	0.58	±0.9051
3.	I’d like to try game meat if it was cheaper.	0.05	±1.4263
4.	If it were widely available in stores, I’d like to try game.	0.04	±1.4242
5.	Eating wild boar meat is synonymous with trichinella infection. (N)	1.02	±1.0702
6.	The nutritional value of wild animal meat is the same as that of farm animals.	−0.41	±1.1301
7.	Game meat is lean.	−0.02	±1.1219
-	Nowadays, hunting is a pastime, not a necessity. (N)	−0.46	±1.3684
8.	Game is meat that has not been sufficiently researched. (N)	0.39	±1.1367
9.	Game meat contains more minerals than livestock meat.	0.14	±0.9627
10.	Game meat is difficult to prepare. (N)	−0.26	±1.0983

Negative statements are marked yellow, and positive statements are marked green.

**Table 2:** Equity and cumulative percentage of variability.

No. of Statement	Eigenvalues	Cumulative % of the Variation
1.	3.0957	30.96
2.	1.9032	49.99
3.	1.0914	60.90
4.	0.9017	69.92
5.	0.8823	78.74
6.	0.5887	84.63
7.	0.5166	89.80
8.	0.4919	94.71
9.	0.4239	98.95
10.	0.1046	100.00

Negative statements are marked yellow, and positive statements are marked green.

**Table 3:** Factor charges after the varimax rotation.

Lp.	Statement	Factor 1	Factor 2	Factor 3	Factor 4
1.	There are health benefits to consuming wild animal meat.	0.715	−0.128	0.324	−0.030
2.	Wild animal meat is heavily contaminated with heavy metals. (N)	−0.131	0.759	−0.068	0.044
3.	I’d like to try game meat if it was cheaper.	0.926	−0.056	0.120	0.077
4.	If it were widely available in stores, I’d like to try game.	0.928	−0.052	0.140	−0.012
5.	Eating wild boar meat is synonymous with trichinella infection. (N)	−0.049	0.768	0.009	0.130
6.	The nutritional value of wild animal meat is the same as that of farm animals.	0.301	0.641	−0.018	−0.318
7.	Game meat is lean.	0.152	0.048	0.859	−0.139
8.	Game is meat that has not been sufficiently researched. (N)	−0.292	0.628	0.005	0.331
9.	Game meat contains more minerals than livestock meat.	0.276	−0.110	0.754	0.251
10.	Game meat is difficult to prepare. (N)	0.079	0.164	0.031	0.867

The numbers in red show which factors were used to assign each statement to a domain. Statements classified to domain 1 are marked in blue, domain 2—orange, domain 3—yellow, and domain 4—gray.

**Table 4:** Cronbach’s alpha values in the assessment of the reliability of domains.

Parameter	Domain 1	Domain 2	Domain 3	Domain 4
Cronbach’s Alpha	0.88	0.62	0.61	-

**Table 5:** Averages and dispersion measures for scale questions and the proposed domains.

No. of Statement	Avg.	SD	Med	QD	Domain						
					No.	Avg.	SD	Med	QD	Q1	Q3
1.	−0.02	1.15	0.00	2.00	1	0.03	1.34	0.00	1.00	−1.00	1.00
3.	0.06	1.43	0.00	2.00							
4.	0.05	1.42	0.00	2.00							
2.	−0.58	0.91	0.00	1.00	2	−0.60	1.09	0.00	1.00	−2.00	0.00
5.	−1.02	1.07	−1.00	2.00							
6.	−0.41	1.13	1.00	1.00							
8.	−0.39	1.14	0.00	1.00							
7.	−0.01	1.12	0.00	1.00	3	0.07	1.05	0.00	0.50	0.00	1.00
9.	0.14	0.96	0.00	1.00							
10.	0.25	1.10	0.00	1.00	4	0.25	1.10	0.00	0.50	0.00	1.00

Negative statements are marked yellow, and positive statements are marked green.

## Data Availability

The data presented in this study are available on request from the corresponding author. The data are not publicly available due to privacy.
